# Plasma Electrolytic Oxidation of Zr-1%Nb Alloy: Effect of Sodium Silicate and Boric Acid Addition to Calcium Acetate-Based Electrolyte

**DOI:** 10.3390/ma15062003

**Published:** 2022-03-08

**Authors:** Veta Aubakirova, Ruzil Farrakhov, Vasily Astanin, Arseny Sharipov, Mikhail Gorbatkov, Evgeny Parfenov

**Affiliations:** Department of Electronic Engineering, Ufa State Aviation Technical University, 12 Karl Marx Street, 450008 Ufa, Russia; frg1982@mail.ru (R.F.); v.astanin@gmail.com (V.A.); arsenyarseny36728@gmail.com (A.S.); mikesg@mail.ru (M.G.); evparfenov@mail.ru (E.P.)

**Keywords:** Zr-1%Nb alloy, plasma electrolytic oxidation, boric acid, medical implants

## Abstract

This work aimed at the development of wear and corrosion resistant oxide coatings for medical implants made of zirconium alloy, by plasma electrolytic oxidation (PEO). The effect of sodium silicate and boric acid addition to calcium acetate electrolyte on the coating properties was studied. Different aspects of the PEO coating were investigated: microstructure, electrochemical and wear behavior, wettability and apatite-forming ability. The resultant coatings consist of a dense inner layer 1.4–2.2 µm thick and a porous outer layer. The total thickness of the coating is 12–20 µm. It was found that the coating contains the tetragonal zirconia (70–95%). The obtained coatings show high corrosion resistance and reduce the surface corrosion current by 1–3 orders of magnitude, depending on the electrolyte additive, compared to the uncoated surface. The addition of boric acid to the electrolyte significantly increases the wear resistance of the coating and reduces the coefficient of friction. In terms of the combination of the coating characteristics, the electrolyte with the addition of the alkali and boric acid is recommended as the most effective.

## 1. Introduction

Zirconium alloys are a promising alternative to titanium alloys in orthopaedic and dental applications due to their better compatibility with magnetic resonance imaging diagnostics because of two-fold lower magnetic susceptibility [1]. The Zr-1Nb alloy has low cytotoxicity, high fatigue strength, and high corrosion resistance, which leads to its investigation as a material for implants [2,3,4]. The Young’s modulus of Zr (92 GPa) is lower than that of Ti (100–110 GPa). Higher elasticity is more favorable for the mechanical compatibility of the implant with the bone [5,6]. These advantages of the zirconium alloy implants can reduce the prrobability of the implant rejection [7,8,9]. However, a simulated body fluid (SBF) causes pitting corrosion on Zr [10]. Moreover, uncoated zirconium has very low bioactivity and does not induce the formation of apatite in the SBF [11]. This is a drawback for permanent medical implants. Therefore, it is important to form a multifunctional coating in order to significantly increase the quality of the implant.

Plasma electrolytic oxidation (PEO) is a method for producing a coating that provides an effective protection of the Zr surface [12,13]. Also, PEO coatings have a porous morphology, which contributes to the fixation of osteoblasts. Moreover, the pore size increases from the substrate to the surface. With such a morphology, the modulus of elasticity changes smoothly from the implant to the interface with the bone. This is favorable for biomechanical compatibility.

An important property of the implant surface is the ability to precipitate hydroxyapatite (HA). For PEO coatings, this issue is considered in papers [14,15,16]. The formation of apatite in SBF provides information on biological activity in vitro [17].

One of the important factors determining the properties of the PEO coating is the composition of the electrolyte. Analysis of the literature shows that the majority of the PEO studies are carried out in phosphate [10,18,19,20,21], silicate [22,23,24,25], and phosphate–silicate electrolytes [21,26,27]. The coatings formed in these electrolytes provide an increase in corrosion resistance and biological activity of the surface. There are fewer works regarding calcium acetate based electrolytes; however, such electrolytes show good results [28,29,30,31] and allow to not only obtain calcium phosphate compounds in the coating, but also stabilize the tetragonal and cubic phases of zirconia due to the presence of Ca oxide [32]. The resultant coatings are porous with a developed bioactive surface.

The addition of boron compounds has minor coverage in the literature for PEO of zirconium alloys. Malayoğlu et al. [25] studied the effect of process duration on the wear and corrosion resistance for the coatings obtained in an electrolyte based on sodium silicate and potassium hydroxide with boric acid. However, this electrolyte did not contain calcium compounds. The promising results of boron compound applications for PEO of other alloys are also known. For example, PEO coatings showed an improvement in morphology, wear resistance, and corrosion resistance on magnesium alloys, if borax [33] and sodium borate [34,35] were added to the electrolyte. The morphology of a PEO coating obtained in an electrolyte with boric acid was also investigated for an aluminium alloy [36].

The study aims to obtain wear and corrosion resistant coatings that are promising for medical implants. This work solves the problem of improving the morphology of the PEO coating on the Zr alloy since, in the mentioned studies, large longitudinal pores appear in the bottom layer of the coating; this can be the cause of low wear resistance; high adhesion and wear resistance are important during screwing in the implant. This work examines the effect of silicate and boron compounds of on the PEO coatings obtained in an electrolyte based on calcium acetate with phosphate and sodium hydroxide to recommend an electrolyte composition with optimal corrosion, wear and apatite forming properties for medical implants.

## 2. Materials and Methods

In this research, we used Zr-1%Nb alloy, the chemical composition of which is provided in Table 1. The samples were cut out of a 0.8 mm thick sheet, to a size of 10 mm × 20 mm. The samples before the formation of the PEO coating were prepared as follows: polished up to P4000 grit SiC paper; washed ultrasonically in distilled water (5 min) and isopropyl alcohol (5 min).

### 2.1. Plasma Electrolytic Oxidation

We used 6-litre tank of electrolyte for PEO process. Temperature of electrolyte was 20 ± 1 °C. It was maintained by heating and cooling system under microcontroller regulation (TRM202, Owen, Russia). Electrolyte conductivity and pH were measured with Anion-4100 (Anion, Novosibirsk, Russia). The electrolyte compositions, corresponding sample codes, and electrolyte conductivities are given in Table 2.

We used the PEO equipment (USATU, Ufa, Russia) in pulsed unipolar mode [37]. The pulse amplitude was 480 V, frequency 700 Hz, duty cycle 26%. The pulse voltage was ramped for 60 s from zero to the setpoint, then, it was kept at a constant level. The treatment time was 10 min.

The automated PEO equipment records the RMS value of current and voltage during the technological process every 1 s. The measurement error of RMS current density values is ±0.03 A∙cm^−2^; the error of RMS voltage value is ±7 V.

### 2.2. Surface Characterization

The coating thickness was measured by DeFelsko Positector 6000 eddy current gauge with an N-type sensor. In addition, cross-section images were analysed. The top view of the PEO coating was studied using JEOL JSM-6490LV scanning electron microscope (SEM) (JEOL, Tokyo, Japan) and by Hitachi Regulus 8220 SEM (Hitachi, Tokyo, Japan). The coating elemental composition was determined using INCAX attachment of JEOL JSM-6490LV by the EDS analysis method.

The surface roughness was measured with the TR-220 profilometer (TIME Group Inc, Beijing, China). The coating porosity was assessed with Image J software from the SEM images following the ASTM E112-10.

The phase composition of the surface layer was characterized by X-ray diffractometer Rigaku Ultima IV (Rigaku, Tokyo, Japan) in CuKα radiation at 40 kV and 40 mA using 0.02 deg. step scan with 2 s exposure, from 25 to 80 degrees 2θ. Further, the XRD spectra were processed using X’Pert Highscore Plus 3.0 (PANalytical B.V., Almelo, The Netherlands) software with PDF2 pattern database; a built-in SemiQuant algorithm was employed to quantify the amounts of the crystalline phases in the coating.

The tribological properties were tested by a pin-on-disc Nanovea TRB-1 tribometer (Nanovea, Inc., Irvine, CA, USA) at a normal load of 2 N against a 6 mm diameter Al_2_O_3_ ball at a room temperature. The sliding speed was 0.1 m/s for a distance of 200 m.

### 2.3. Electrochemical Tests

We used Ringer’s solution at 37 °C for electrochemical tests performed by the P-5X (Elins, Moscow, Russia) electrochemical station. The composition of 1 dm^3^ Ringer solution was declared by the manufacturer (Solopharm, Saint Petersburg, Russia): NaCl (8.6 g), KCl (0.3 g), CaCl_2_·6H_2_O (0.25 g) in distilled water. The potentiodynamic polarization (PDP) was performed in ±600 mV range against the open circuit potential (OCP) at 0.25 mV/s scan rate. A silver chloride electrode filled with 3.5 M KCl was used as a reference. The counter electrode was a graphite rod. The PDP results were processed using Tafel’s method. The polarization resistance *R_p_* was calculated from the slope of the polarization curve ±10° mV around the free corrosion potential. The electrochemical impedance spectroscopy (EIS) measurement was conducted by applying voltage with a magnitude of ±10 mV around the OCP in a frequency range of 100 kHz to 10 mHz. The EIS results were analysed using ZView software from Scribner Associates.

### 2.4. Apatite Forming Ability Test 

The SBF was prepared by dissolving proper amounts of NaCl (7.996 g), NaHCO_3_ (0.350 g), KCl (0.224 g), K_2_HPO_4_·3H_2_O (0.228 g), MgCl_2_·6H_2_O (0.305 g), CaCl_2_ (0.278 g), Na_2_SO_4_(0.071 g), (CH_2_OH)_3_CNH_2_ (6.057 g) and 1M-HCl (38 mL) in a distilled water according to Kokubo [17] for 1 dm^3^ solution. The tests were conducted by soaking the samples at 37 ± 1 °C in the SBF for 30 days. The specific weight change was measured using A&D GR-200 analytical balance. We detected the weight of the samples before (m_1_) and after (m_2_) SBF test to calculate the specific weight change:

∆m = (m_2_ − m_1_)∙S^−1^ (1)

where S is the sample surface area.

The value of the Ca/P ratio is an important factor in the process of bone tissue ingrowth. To determine Ca/P value, the weight percentages were converted to atomic percentages and the ratio was found. The Ca/P ratio is 1.67 for the hydroxyapatite, the chemical formula of which is Ca_10_(PO_4_)_6_(OH)_2_ [38].

## 3. Results and Discussion

### 3.1. Process Characteristics for PEO of Zirconium Alloy

**Figure 1:**
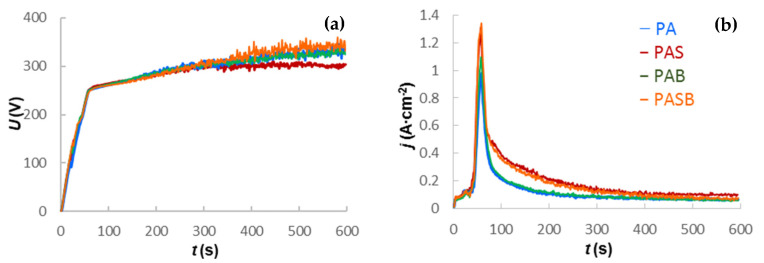
Electrical characteristics for the PEO process of Zr1%Nb samples: (**a**) RMS voltage; (**b**) RMS current density.

Figure 1 shows how the RMS voltage and RMS current density changed during the PEO for PA, PAS, PAB, and PASB samples. In the time range from 0 to 50 s, the samples primarily undergo an anodizing process. When the ignition voltage of the microdischarges is reached, there is a sharp peak and an exponential decrease in the current density (in the time interval from 50 to 350 s). From 350 to 600 s, the current density almost reaches the steady state. The differences in the current density values can be correlated with the electrolyte specific conductivities (Table 2).

The RMS voltage during the treatment increases with the growth of the coating thickness [39] because the system of pores formed as a result of the action of microdischarges influences both the conductivity and effective capacitance of the coating. This affects the shape of the fall transients of the voltage pulses, and the RMS value itself [40]. The lowest final RMS voltage for the PAS sample suggests the thickest inner layer and the smallest overall coating thickness. In turn, the highest RMS value for the PASB sample suggests the thinner inner layer and the largest overall coating thickness.

### 3.2. Surface Morfology

**Figure 2:**
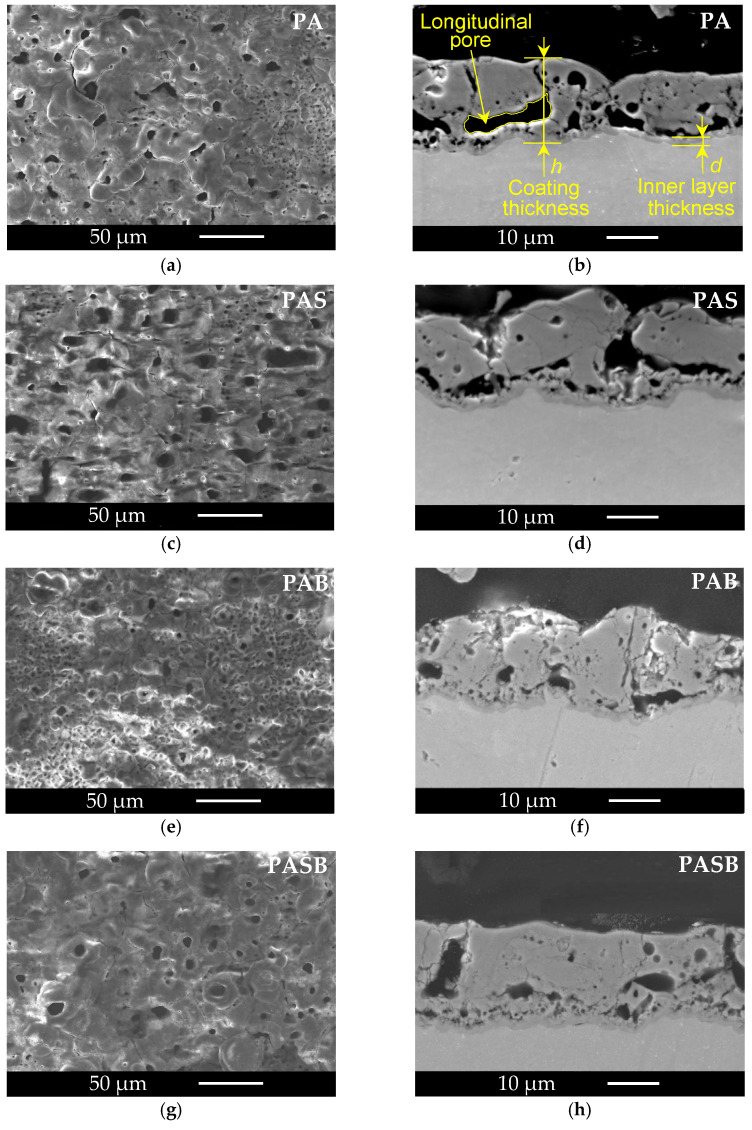
Surface morphologies of the PEO coatings produced in (**a**) PA, (**c**) PAS, (**e**) PAB, (**g**) PASB electrolytes, and cross sections of the coatings (**b**) PA, (**d**) PAS, (**f**) PAB, (**h**) PASB.

The surface morphologies and cross-sections of the coatings are shown in Figure 2. The coatings consist of a dense inner layer with a thickness d = 1.4–2.2 µm and a porous outer layer. Table 3 shows the values of the coating thickness and parameters of the surface roughness. The addition of the sodium silicate leads to the fact that the pore system becomes more uniform, large pores with a size of 7.5 ± 2.4 µm prevail on the surface (Figure 2c,g); while, without the addition of the sodium silicate, areas with small pores 1.3 ± 0.5 µm are observed between the large pores (Figure 2a,e). The difference in the surface topography due to the different pore distributions can be described by the roughness parameter RPc (the number of profile elements per 1 centimeter of length that are above the set limit and immediately after that below the set limit) [41]. The RPc of the surface of the coating formed with the sodium silicate is 7–20% less than that of the coating obtained in a similar electrolyte without the sodium silicate. In addition, if we consider the vertical components of the roughness: average and maximum roughness Ra and Rmax, respectively, the smoothest surface belongs to the PAS sample, while the roughest–to the PASB. The values of the parameters of the vertical roughness correlate with the coating thickness. Moreover, the suggestions from the RMS voltage evolution are supported with the values from Table 3.

Let us examine the coating cross-sections. The addition of boric acid resulted in a significant reduction in longitudinal pores in the lower part of the coating. PAB and PASB coatings appear denser. Such an improvement in the quality of the coating can be explained by the described effects of the addition of boron compounds, oxides, and calcium borates in the ceramic industry [42,43] where boron compounds are used to lower the melting point and viscosity of the glassy phase. During the PEO, micro-discharges cause local temperature rise to more than 3000 K [44,45]. The explosion of a gas bubble with the microdischarge leads to splashing of a melt of metal and oxides. In the presence of the boron compounds, the melting range of the coating is extended and the cavity in the coating thickness, formed by the gas bubble, has more time to fill with the melt. A similar decrease in the pore size of the coating was observed during the PEO of a magnesium alloy with the addition of Na_2_B_4_O_7_ to the electrolyte [35].

### 3.3. Elemental and Phase Composition of the PEO Coatings 

Table 4 presents the results of the elemental EDS analysis. All PEO coatings contain O, Zr, Nb, and Ca. In addition, samples PAS and PASB contain Si, which migrrated into the coating structure from the electrolyte containing the sodium silicate. The sample PAB contains phosphorus, the amount of which slightly exceeds the resolution threshold. The amount of O correlates with the coating thickness (Table 3).

**Figure 3:**
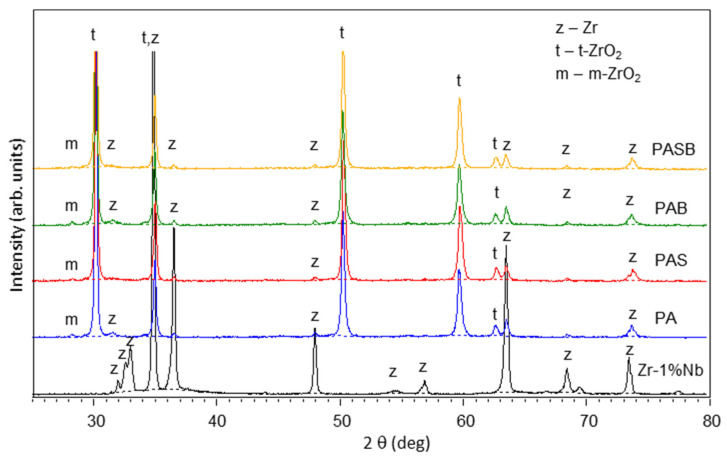
X-ray diffractograms of the substrate, and PA, PAS, PAB, PASB-coatings.

The phase structure of the PEO coatings on the zirconium alloy is illustrated in Figure 3. Table 5 shows the evaluation of the crystalline phases content from the XRD; the phases of monoclinic-ZrO_2_ (m-ZrO_2_) (JCPDS Card No: 01-078-0047 ) and tetragonal-ZrO_2_ (t-ZrO_2_) (JCPDS Card No: 98-009-2543) were detected. m-ZrO_2_ was detected in minor quantities, in contrast to the large amount of the t-ZrO_2_. Since t-ZrO_2_ is a high-temperature phase, the transformation of the monoclinic phase into the tetragonal one occurs at a temperature of 1170 °C [46] under the action of microdischarges. The presence of the tetragonal phase at room temperature is explained by its stabilization due to the effect of the calcium oxide [29]. Silicon can be in an amorphous form since no peaks of silicon-containing phases were found in the X-ray diffractograms. The X-ray showed no individual peaks of the calcium oxide either. Therefore, calcium may be contained in an amorphous state and/or as a partially stabilized compound Ca_x_Zr_x_O_x_ [14,47], calcium borate Ca_3_BO_3_, which peaks coincide with the peaks of ZrO_2_, so they cannot be reliably quantified.

### 3.4. SBF Test

**Figure 4:**
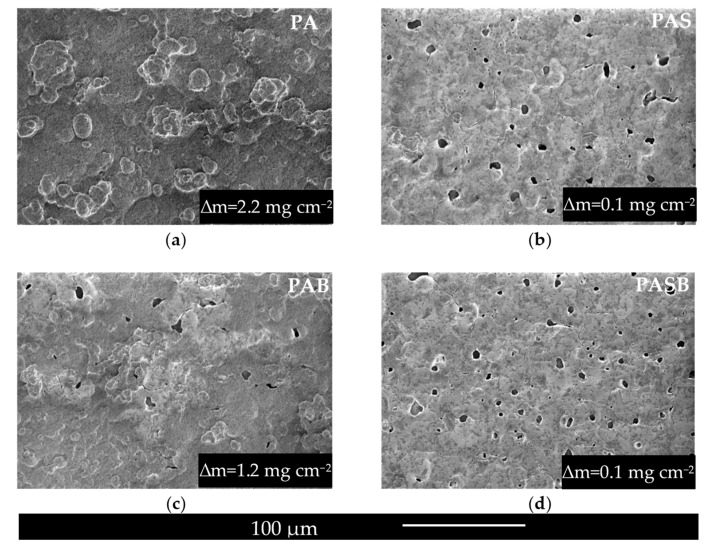
Surface morphologies of the PEO coatings produced in (**a**) PA, (**b**) PAS, (**c**) PAB, (**d**) PASB electrolytes after the SBF test.

Figure 4 shows the surface of PEO coatings after the SBF-test. The outgrowths of the secondary apatite observed on the surface provides information on biological activity under in vitro conditions, as noted elsewhere [15,47,48]. The entire surface and pores of the PA sample were completely covered by the apatite. The apatite settled on the surface of the PAB sample without covering the large pores. There was much less apatite on the surface of the PAS and PASB samples, and rare accumulations of HA were observed.

The weight gain ∆m values presented in Figure 4 suggest that the sample PA has the highest ∆m. The ∆m of the PAS and PASB samples have small values close to zero. The lowest apatite forming ability of the PAS and PASB samples can be explained by interrelated reasons: sample morphology (smooth surface areas between large pores, while in the case of PA and PAB these areas are covered with a system of small pores) and hydrophobic type of surface. 

**Figure 5:**
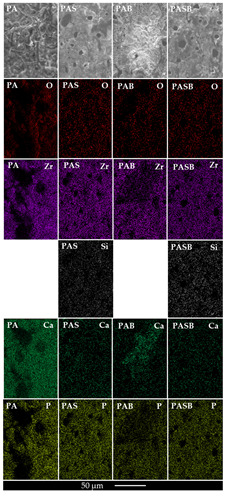
Elemental mapping of the PA, PAS, PAB, PASB-coatings after SBF-test.

Table 6 present the results of the elemental EDS analysis after the SBF test. In addition, the elemental distribution maps for the surfaces of PA, PAS, PAB, and PASB samples are presented in Figure 5. Elemental analysis showed an increase in calcium and phosphorus elements on the PA and PAB surfaces. The largest amounts of Ca and P were found on the surface of the PA sample, which is consistent with the ∆m data and the image in the micrographs. In the silicon-containing coatings, the calcium content decreased after exposure to the Kokubo solution. Perhaps some of the calcium was in amorphous compounds, which further dissolved. 

The Ca/P ratio of the PAB sample is higher, which may indicate the formation of a mixture of the hydroxyapatite and amorphous calcium compounds. The Ca/P ratio of the PA sample corresponds to the Ca/P ratio of hydroxyapatite. As shown elsewhere [49], the ratio higher than 1.67 is also favorable for the induction of the osteogenesis; therefore, the PAB sample is promising for implant applications.

**Figure 6:**
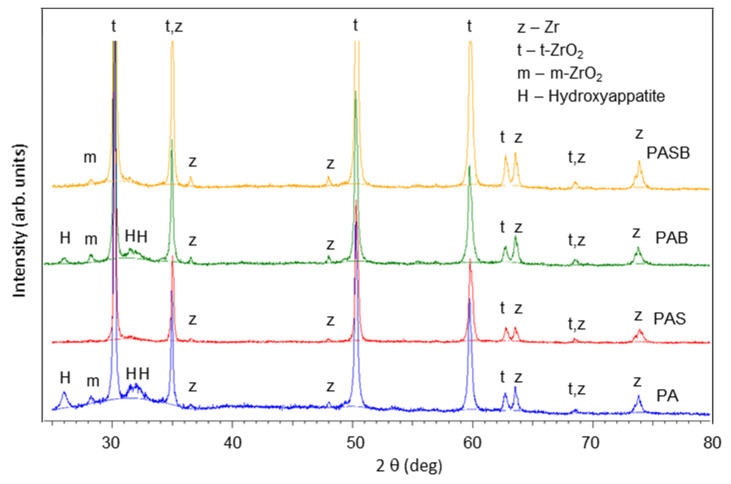
X-ray diffractogram of the PA, PAS, PAB, PASB-coatings after the SBF test.

The X-ray diffractograms of the samples after the SBF test are shown in Figure 6. Table 7 shows the evaluation of the crystalline phases content from the XRD. It can be seen from the X-ray diffractograms that the PA, PAB samples have additional peaks identified as hydroxyapatite (JCPDS Card No: 01-072-1243), which precipitated from the SBF solution. This phenomenon correlates with the distribution of elements Ca and P in the coating presented in Figure 5.

### 3.5. Wettability Tests of the Coatings

**Figure 7:**
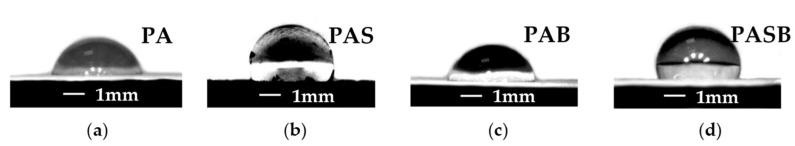
The surface contact angle measurements of samples (**a**) PA, (**b**) PAS, (**c**) PAB, and (**d**) PASB.

The test images are shown in Figure 7. The contact angle of PA (75° ± 3°) and PAB (78° ± 4°), demonstrates their hydrophilic nature. Both contact angles are less than 90°. The PAS and PASB samples show hydrophobic properties with identical contact angles of 104 ± 5°. The greater number of small pores in the PA and PAB samples contributes to a lower contact angle than in the PAS and PASB samples. The influence of morphology seems to be a more likely reason for the difference in the contact angles than the assumption of the silicon presence in the coating, despite the fact that in a known work [27], Si-containing coatings showed hydrophilic properties.

### 3.6. Electrochemical Behavior of the Uncoated and PEO Coated Samples

**Figure 8:**
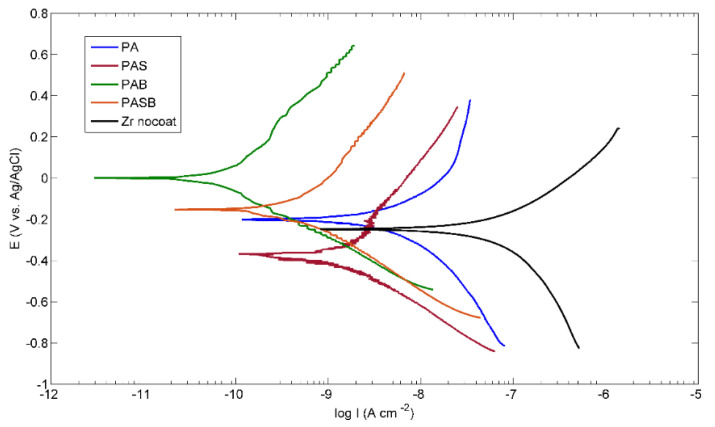
Polarization curves in Ringer’s solution for the uncoated and PEO-coated samples.

Figure 8 shows the PDP curves of the uncoated sample and the samples after the PEO treatment. The results of the calculated corrosion properties for all the studied samples are presented in Table 8. From the values of the free corrosion potentials E*_corr_* of the samples, it follows that the PEO treatment in the PAS electrolyte makes the sample surface less noble. This is clear from the lower value of the potential of the free corrosion in comparison with the untreated sample. Treatment in PA, PAB, and PASB electrolytes leads to surface passivation, which indicates a higher E*_corr_* value in comparison with the untreated sample, while the PAB sample demonstrated the noblest potential.

**Figure 9:**
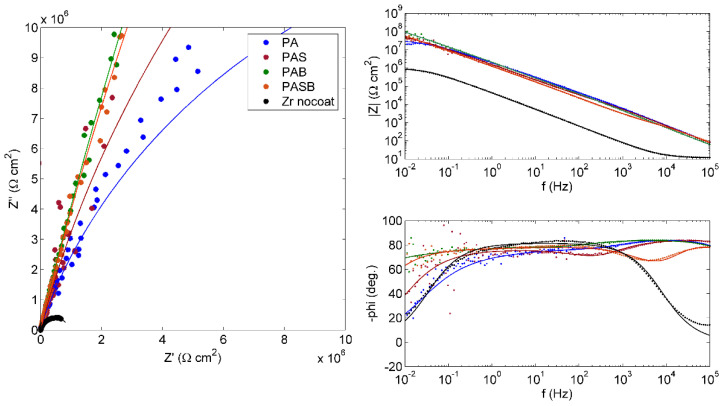
Nyquist and Bode plots for the EIS of the uncoated and PEO-coated samples in Ringer’s solution.

**Figure 10:**
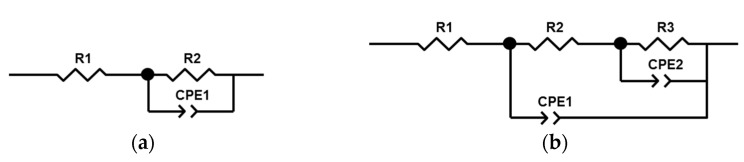
Equivalent circuits used for the EIS results fitting: (**a**) Zr substrate; (**b**) PEO-coated samples.

A noticeable decrease in the corrosion current i*_corr_* of the treated samples compared to the untreated sample can be seen. The best corrosion resistance is demonstrated by the PAB sample, where i*_corr_* is less than that of the untreated sample by more than two orders of magnitude. The values of the polarization resistances R_p_ are in a good agreement with the data of the corrosion current. Figure 9 shows the EIS results in the form of the Nyquist and Bode plots for the uncoated sample and the samples after the PEO treatment. The EIS results were approximated by the equivalent circuits shown in Figure 10a,b.

The impedance of the untreated sample was approximated by an equivalent circuit with a single time constant (Figure 10a). This Randles circuit is the main one for the modelling of the electrochemical processes. The impedances of the samples after the PEO treatment were approximated by a ladder circuit (Figure 10b), which is well suited for modelling the impedance of two-layered coatings [50]. The PEO treatment in all the electrolytes increases the impedance modulus |Z| by more than an order of magnitude in the entire range of scanned frequencies, which can be seen from the Bode diagram.

The results of calculating the parameters of equivalent circuits are presented in Table 9. The electrolyte resistance was R_1_ = 9.8 ± 1.9 Ω cm^2^ for all samples. In the circuit with one time constant, element R_2_ is the charge transfer resistance. The value of the CPE1-Q element can be considered as an evaluation of the double layer capacitance (provided that CPE1-n is close enough to unity), and it correlates well with the thickness of the defect-free natural layer h ~ 1/CPE1-Q. A higher CPE1-Q value indicates a thinner layer.

The EIS spectra of the treated samples show two-time constants; this indicates two relaxation processes in the two-layered PEO coating. The pairs of elements R2-CPE1 and R3-CPE2 indicate the resistance and "capacitance" of the outer porous layer and the inner compact layer, respectively. According to the SEM photographs shown in Figure 2, the coating obtained in the PAB electrolyte appears to be the more dense, and with the least observed number of defects. As a result, this coating possesses the best protective properties, which can be seen from the values of the calculated parameters of the equivalent circuit and the corrosion resistance calculated at minimum frequency |Z| _f → 0_ = R1 + R2 + R3 ≈ R3 = 1.58·× 10^9^ Ω·cm^2^.

### 3.7. Tribological Tests

**Figure 11:**
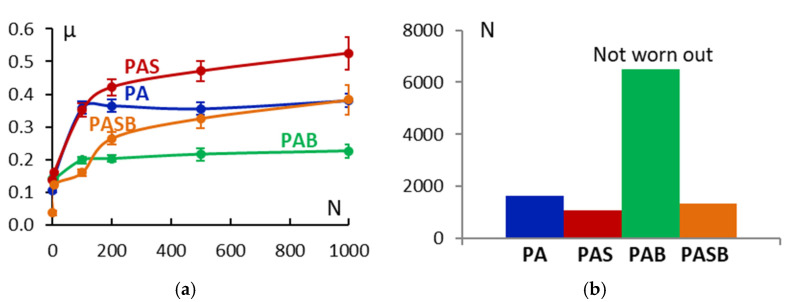
Change in the coefficient of friction depending on the number of revolutions (**a**) and the number of revolutions before destruction of the coating (**b**) for the PEO-coated samples PA, PAS, PAB, and PASB.

**Figure 12:**
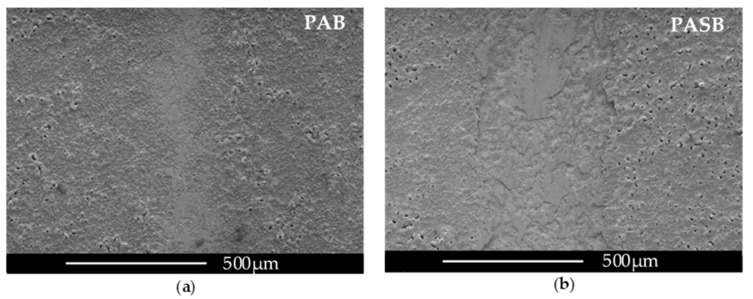
Micrograph of a wear track from a rotating Al_2_O_3_ ball with a diameter of 6 mm after passing 200 m at a load of 2 N for a PAB sample (**a**), and PASB sample (**b**).

Figure 11 shows the change in the coefficient of friction µ with revolutions N from 0 to 1000. µ increases during the first 100 revolutions for all the samples, as follows from Figure 11a. Further, the coefficient of friction grows for the samples PAS and PASB. For the PA and PAB samples µ remains at a level of 0.38 ± 0.04 and 0.23 ± 0.04, respectively. Figure 11b shows the number of revolutions until the spinning ball touches the metal substrate when the coating is abraded. The PAB sample showed the best wear resistance and the lowest µ value; the coating did not break during the test. The PAS sample had the worst wear resistance. Micrographs of the spinning ball wear track are shown in Figure 12.

When comparing the samples, it can be noted that the addition of boric acid to the electrolyte reduces the coefficient of friction by a factor of 1.3–1.7. The addition of sodium silicate, on the other hand, increases the coefficient of friction by a factor of 1.3–1.7.

## 4. Conclusions

PEO coatings were produced on Zr-1%Nb alloy in four electrolytes based on sodium phosphate, sodium hydroxide, and calcium acetate-based solutions with boric acid and sodium silicate additions.

- The boric acid addition decreases pore size in the coating thickness, making the coating denser. As a result of improving the morphology, the corrosion and wear resistance of the coating increased significantly.
- It is assumed that within the PEO process mechanism, the boron compounds reduce the melting temperature and viscosity of the oxide film during microdischarge events. In this case, the solidification of the melt occurs rather slowly, and the cavity-pore in the coating formed by the gas bubble has more time to fill up. Thus, a reduction in pore size is achieved.
- The addition of sodium silicate to the electrolyte composition leads to coarsening of the pore size on the surface of the coating and an increase in the coefficient of friction. A less wear-resistant surface was obtained compared to experiments without silicate additives. It should be noted that this additive increases the hydrophobicity of the surface.
- The best precipitation of hydroxyapatites from the SBF was observed in the experiment in the PA and PAB electrolytes. On the surface of the coating obtained in an electrolyte with the addition of silicate, the amount of hydroxyapatites was small and not captured by the SEM method.

As a result of the assessment, the following electrolytes were identified as promising for further in vitro and in vivo tests of the coatings:

- PAB, which provides the highest corrosion and wear resistance;
- PA, which provides the highest amount of calcium phosphate compounds and a high coefficient of friction, favorable for reliable contact of the implant with the bone tissue.

## Figures and Tables

**Table 1:** Chemical composition of Zr-1Nb alloy (weight%).

Nb	O	Hf	Fe	Ca	C	Ni	Cr	Si	Zr
1.10	0.10	0.05	0.05	0.03	0.02	0.02	0.02	0.02	Balance

**Table 2:** Sample codes corresponding to electrolyte compositions.

Sample Code	Electrolyte Composition	Electrolyte Conductivity (S cm^−1^)	pH
PA	15 g L^−1^ Na_3_PO_4_∙12 H_2_O + 25 g L^−1^ Ca(CH_3_COO)_2_·1 H_2_O + 1 g L^−1^ NaOH	14.77 ± 0.05	10.5 ± 0.1
PAS	15 g L^−1^ Na_3_PO_4_∙12 H_2_O + 25 g L^−1^ Ca(CH_3_COO)_2_·1 H_2_O + 1 g L^−1^ NaOH + 10 g L^−1^ Na_2_SiO_3_∙12 H_2_O	15.77 ± 0.06	11.1 ± 0.1
PAB	15 g L^−1^ Na_3_PO_4_∙12 H_2_O + 25 g L^−1^ Ca(CH_3_COO)_2_·1 H_2_O + 1 g L^−1^ NaOH + 1 g L^−1^ H_3_BO_3_	14.96 ± 0.04	8.4 ± 0.1
PASB	15 g L^−1^ Na_3_PO_4_∙12 H_2_O + 25 g L^−1^ Ca(CH_3_COO)_2_·1 H_2_O + 1 g L^−1^ NaOH + 10 g L^−1^ Na_2_SiO_3_∙12 H_2_O + 1 g L^−1^ H_3_BO_3_	16.15 ± 0.07	9.9 ± 0.1

**Table 3:** Morphological characteristics of the PEO coatings.

Sample Code	Coating Thickness *h*, µm	Inner Layer Thickness, *d*, µm	Roughness		
			Ra, µm	Rmax, µm	RPc, Pieces/cm
PA	14.7 ± 0.8	1.8 ± 0.3	1.66 ± 0.2	13.26 ± 1.67	147 ± 9
PAS	13.9 ± 1.1	2.2 ± 0.2	1.51 ± 0.18	11.22 ± 0.25	118 ± 6
PAB	15.7 ± 2.6	1.7 ± 0.3	1.62 ± 0.07	14.03 ± 0.64	130 ± 11
PASB	17.5 ± 2.0	1.7 ± 0.2	1.76 ± 0.13	15.88 ± 0.74	122 ± 11
Zr nocoat	-	-	0.052 ± 0.003	0.61 ± 0.10	1 ± 1

**Table 4:** The elemental composition of samples PA, PAS, PAB, and PASB obtained by EDS.

Sample Code	Content of the Elements in the Coating (wt%)					
	O	Zr	Si	Ca	Nb	P
PA	24.4 ± 0.7	68.8 ± 2.3	-	5.6 ± 0.4	1.2 ± 0.3	-
PAS	24.7 ± 0.7	66.6 ± 1.8	2.13 ± 0.3	5.3 ± 0.5	1.2 ± 0.3	-
PAB	27.5 ± 0.8	62.8 ± 1.6	-	8.1 ± 0.4	1.1 ± 0.3	0.5 ± 0.3
PASB	26.2 ± 0.7	61.8 ± 1.5	2.8 ± 0.4	8.6 ± 0.4	0.7 ± 0.3	-

**Table 5:** Evaluation of the crystalline phases content (wt%) in the PEO coatings by the X-ray diffraction analysis.

Sample Code	Content of the Crystalline Phases in the Coating (wt%)	
	t-ZrO_2_	m-ZrO_2_
PA	94 ± 6	6 ± 4
PAS	72 ± 5	28 ± 2
PAB	93 ± 6	7 ± 1
PASB	83 ± 6	17 ± 1

**Table 6:** The elemental composition of samples PA, PAS, PAB, and PASB after the SBF test.

Sample Code	Content of the Elements in the Coating (wt%)						
	O	Zr	Si	Ca	Nb	P	Ca/P
PA	41.1 ± 0.8	11.1 ± 1.0	-	32.1 ± 1.9	0.2 ± 0.1	15.5 ± 0.8	1.6 ± 0.2
PAS	24.7 ± 0.9	66.6 ± 1.1	2.6 ± 0.4	5.0 ± 0.2	1.1 ± 0.2	-	-
PAB	26.3 ± 0.8	62.4 ± 1.1	-	8.3 ± 0.3	1.0 ± 0.2	2.0 ± 0.3	3.2 ± 0.7
PASB	28.6 ± 0.7	63.2 ± 0.8	2.2 ± 0.2	5.0 ± 0.3	1.0 ± 0.2	-	-

**Table 7:** Evaluation of the crystalline phases content (wt.%) in PEO coatings after the SBF test by the X-ray diffraction analysis.

Sample Code	Content of the Crystalline Phases in the Coating (wt%)		
	t-ZrO_2_	m-ZrO_2_	Hydroxyapatite
PA	68 ± 5	7 ± 2	25 ± 2
PAS	79 ± 5	21 ± 2	-
PAB	75 ± 5	7 ± 1	18 ± 2
PASB	85 ± 6	15 ± 2	-

**Table 8:** Results of potentiodynamic corrosion tests in Ringer’s solution for the uncoated and PEO-coated samples.

Sample Code	*E_corr_* (V vs. Ag/AgCl)	*i_corr_* (nA·cm^−2^)	*R_p_* (MΩ cm^2^)
Zr	−0.248 ± 0.03	51.7 ± 10.4	1.15 ± 0.231
PA	−0.201 ± 0.02	7.58 ± 1.90	13.2 ± 9.13
PAS	−0.381 ± 0.004	1.42 ± 0.43	62.7 ± 28.3
PAB	0.000 ± 0.01	0.086 ± 0.031	781 ± 277
PASB	−0.152 ± 0.02	0.63 ± 0.36	132 ± 76.7

**Table 9:** EIS fit results of the parameters of the equivalent circuits for the uncoated and PEO-coated samples tested in Ringer’s solution.

Sample Code	Parameters of the Equivalent Circuits					
	R2 (Ω cm^2^)	R3 (Ω cm^2^)	CPE1-Q (F^n−1^·cm^−2^)	CPE1-n	CPE2-Q (F^n−1^·cm^−2^)	CPE2-n
Zr	9.36 × 10^5^ ± 1.11·× 10^4^	-	4.41·× 10^−6^ ± 2.61·× 10^−8^	0.91 ± 0.001	-	-
PA	4.48·× 10^4^ ± 1.70·× 10^4^	3.92·× 10^7^ ± 1.30·× 10^6^	3.98·× 10^−8^ ± 3.61·× 10^−9^	0.95 ± 0.008	8.04·× 10^−8^ ± 3.76·× 10^−9^	0.69 ± 0.009
PAS	3.43·× 10^4^ ± 9.32·× 10^3^	7.83·× 10^7^ ± 6.57·× 10^6^	4.86·× 10^−8^ ± 6.90·× 10^−9^	0.93 ± 0.013	9.01·× 10^−8^ ± 7.41·× 10^−9^	0.78 ± 0.009
PAB	2.59·× 10^5^ ± 9.33·× 10^4^	1.58·× 10^9^ ± 8.44·× 10^8^	6.02·× 10^−8^ ± 2.77·× 10^−9^	0.93 ± 0.004	4.37·× 10^−8^ ± 2.78·× 10^−9^	0.71 ± 0.012
PASB	1.25·× 10^3^ ± 1.31·× 10^2^	2.47·× 10^8^ ± 2.46·× 10^7^	1.41·× 10^−7^ ± 7.19·× 10^−9^	0.83 ± 0.002	2.83·× 10^−8^ ± 6.91·× 10^−9^	0.97 ± 0.019

## Data Availability

Not applicable.
